# Disclosing the Antifungal Mechanisms of the Cyclam Salt H_4_[H_2_(^4-CF3^PhCH_2_)_2_Cyclam]Cl_4_ against *Candida albicans* and *Candida krusei*

**DOI:** 10.3390/ijms25105209

**Published:** 2024-05-10

**Authors:** Inês Costa, Inês Lopes, Mariana Morais, Renata Silva, Fernando Remião, Rui Medeiros, Luís G. Alves, Eugénia Pinto, Fátima Cerqueira

**Affiliations:** 1 UCIBIO—Applied Molecular Biosciences Unit, REQUIMTE, Laboratory of Toxicology, Department of Biological Sciences, Faculty of Pharmacy, University of Porto, Rua de Jorge Viterbo Ferreira nº 228, 4050-313 Porto, Portugal; inessilvacosta@hotmail.com (I.C.); rsilva@ff.up.pt (R.S.); remiao@ff.up.pt (F.R.); 2 Associate Laboratory i4HB—Institute for Health and Bioeconomy, Faculty of Pharmacy, University of Porto, 4050-313 Porto, Portugal; 3 Molecular Oncology and Viral Pathology Group, Research Center of IPO Porto (CI-IPOP)/RISE@CI-IPOP (Health Research Network), Portuguese Oncology Institute of Porto (IPO Porto)/Porto Comprehensive Cancer Center (Porto.CCC), 4169-007 Porto, Portugal; mariaineslopes180@gmail.com (I.L.); mariana.gomes.morais@ipoporto.min-saude.pt (M.M.); ruimedei@ipoporto.min-saude.pt (R.M.); fatimaf@ufp.edu.pt (F.C.); 4 School of Health, Polytechnic Institute of Porto, Rua Dr. António Bernardino de Almeida 400, 4200-072 Porto, Portugal; 5 ICBAS, Abel Salazar Institute for the Biomedical Sciences, University of Porto, Rua Jorge de Viterbo Ferreira 228, 4050-313 Porto, Portugal; 6 FP-I3ID, FP-BHS, GIT-LoSa, University Fernando Pessoa, Praça 9 de Abril 349, 4249-004 Porto, Portugal; 7 Faculty of Health Sciences, University Fernando Pessoa, Rua Carlos da Maia 296, 4200-150 Porto, Portugal; 8 Centro de Química Estrutural—Institute of Molecular Sciences, Associação do Instituto Superior Técnico para a Investigação e Desenvolvimento, Av. António José de Almeida nº 12, 1000-043 Lisboa, Portugal; luis.g.alves@tecnico.ulisboa.pt; 9 Laboratory of Microbiology, Biological Sciences Department, Faculty of Pharmacy of University of Porto, Rua Jorge de Viterbo Ferreira 228, 4050-313 Porto, Portugal; 10 CIIMAR/CIMAR, Interdisciplinary Centre of Marine and Environmental Research, Terminal de Cruzeiros do Porto de Leixões, 4450-208 Matosinhos, Portugal

**Keywords:** yeasts, mechanism of action, metabolic viability, mitochondrial function, cyclam salt

## Abstract

Mycoses are one of the major causes of morbidity/mortality among immunocompromised individuals. Considering the importance of these infections, the World Health Organization (WHO) defined a priority list of fungi for health in 2022 that include *Candida albicans* as belonging to the critical priority group and *Pichia kudriavzevii* (*Candida krusei*) to the medium priority group. The existence of few available antifungal drugs, their high toxicity, the acquired fungal resistance, and the appearance of new species with a broader spectrum of resistance, points out the need for searching for new antifungals, preferably with new and multiple mechanisms of action. The cyclam salt H_4_[H_2_(^4-CF3^PhCH_2_)_2_Cyclam]Cl_4_ was previously tested against several fungi and revealed an interesting activity, with minimal inhibitory concentration (MIC) values of 8 µg/mL for *C. krusei* and of 128 µg/mL for *C. albicans*. The main objective of the present work was to deeply understand the mechanisms involved in its antifungal activity. The effects of the cyclam salt on yeast metabolic viability (resazurin reduction assay), yeast mitochondrial function (JC-1 probe), production of reactive oxygen species (DCFH-DA probe) and on intracellular ATP levels (luciferin/luciferase assay) were evaluated. H_4_[H_2_(^4-CF3^PhCH_2_)_2_Cyclam]Cl_4_ induced a significant decrease in the metabolic activity of both *C. albicans* and *C. krusei*, an increase in Reactive Oxygen Species (ROS) production, and an impaired mitochondrial function. The latter was observed by the depolarization of the mitochondrial membrane and decrease in ATP intracellular levels, mechanisms that seems to be involved in the antifungal activity of H_4_[H_2_(^4-CF3^PhCH_2_)_2_Cyclam]Cl_4_. The interference of the cyclam salt with human cells revealed a CC_50_ value against HEK-293 embryonic kidney cells of 1.1 μg/mL and a HC_10_ value against human red blood cells of 0.8 μg/mL.

## 1. Introduction

Fungal infections are a public health problem, related with infections that can range from mild superficial to systemic and opportunistic diseases, with a high degree of associated mortality [1,2]. The emergence of new species, the rise of resistance, and the increase in the number of immunosuppressed people susceptible to serious infections, among other factors, are leading health and research institutions to become more concerned about this type of infections [1,2]. In 2022, the World Health Organization (WHO) drew up a document identifying some priority fungi, including *Candida albicans* (critical group) and *Pichia kudriavzevii* (*Candida krusei*) (medium group) [3]. One of the main factors considered for the classification was the antimicrobial resistance. Concern about antimicrobial resistance is also reflected in the WHO’s One Health approach in which the human–animal-environment trilogy is an interdependent determinant of the health of all others, namely human health [4].

One of the strategies to try to overcome the problem of antifungal resistance is the discovery of new molecules with antifungal activity and the potential to be introduced into therapy, either as monotherapy or in combination with conventional antifungal drugs [5,6].

**Figure 1:**
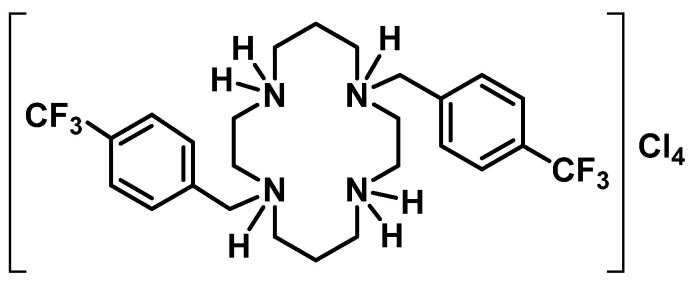
Chemical structure of H_4_[H_2_(^4-CF3^PhCH_2_)_2_Cyclam]Cl_4_ (**1**).

The antifungal activity of the cyclam salt H_4_[H_2_(^4-CF3^PhCH_2_)_2_Cyclam]Cl_4_ (compound **1**), depicted in Figure 1, was previously studied by some of us and showed the ability to inhibit the growth of *C. krusei* and *C. albicans* [7] and to interfere with the yeasts virulence factors, as catalase activity, biofilm formation and *C. albicans* dimorphic transition [8].

Mitochondria are responsible for yeast metabolism and their dysfunction, caused by drugs, influences viability, proliferation, virulence and drug resistance [9,10,11,12]. Thus, mitochondria are currently considered a target for the development of antifungal drugs [9,11,12]. The effect of antifungal drugs on mitochondria may include increased permeability, adenosine triphosphate (ATP) alterations, increased reactive oxygen species (ROS) production and induction of apoptosis [12].

For this reason, the interference of compound **1** on *C. krusei* and *C. albicans* mitochondria was studied to elucidate its mechanism of antifungal action.

## 2. Results

### 2.1. Effects on Yeasts’ Metabolic Activity―Rezazurin Reduction Assay

**Figure 2:**
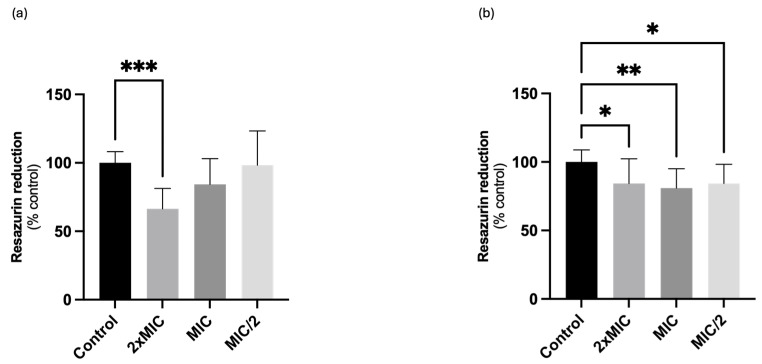
Effects of different concentrations of compound **1** on the metabolic activity of (**a**) *Candida krusei* ATCC 6258 and (**b**) *Candida albicans* ATCC 10231. Results are presented as Mean + SD from 4 or 5 independent experiments, performed in duplicate. * *p* < 0.05; ** *p* ˂ 0.01; *** *p* < 0.001. MIC, minimal inhibitory concentration.

To evaluate a possible interference of compound **1** in the metabolic activity of both *C. krusei* and *C. albicans*, the resazurin (REZ) reduction assay was performed. As observed in Figure 2, the compound significantly reduced *C. krusei* metabolic activity when tested at 16 µg/mL (2× MIC), with a value of 66.4% when compared with control yeasts (100%). Regarding *C. albicans*, compound **1** was able to reduce the metabolic activity in all tested concentrations, (256 (2× MIC), 128 (MIC) and 64 (MIC/2) µg/mL), significantly decreasing REZ reduction to 84.4%, 80.9% and 84.3%, respectively (Figure 2).

### 2.2. Effects on Yeasts’ Mitochondrial Membrane Potential―JC-1 Assay

**Figure 3:**
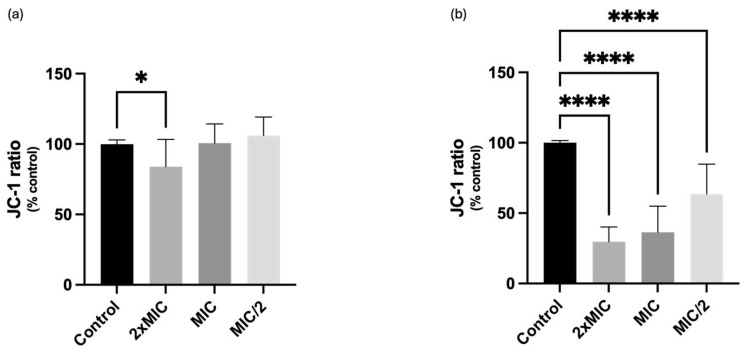
Effects of different concentrations of compound **1** on the mitochondrial membrane potential (ΔΨm) of (**a**) *Candida krusei* ATCC 6258 and (**b**) *Candida albicans* ATCC 10231, evaluated using the JC-1 dye. Results are presented as Mean + SD from 4 or 5 independent experiments, performed in duplicate. * *p* < 0.05; **** *p* ˂ 0.0001. MIC, minimal inhibitory concentration.

The capability of compound **1** to interfere with yeasts mitochondria was evaluated trough the JC-1 assay, which allows one to evaluate the mitochondrial membrane potential [13]. JC-1 can selectively enter the mitochondria and reversibly change its colour from red to green as the membrane potential decreases. At a high concentration of JC-1 (when ΔΨm is also high), the dye aggregates, yielding a red coloured emission. On the other hand, when JC-1 is in a low concentration (when ΔΨm is low), it produces a green fluorescence. Therefore, the mitochondrial depolarization is indicated by a decrease in the red/green fluorescence intensity ratio. Indeed, the decrease in the aggregate fluorescent count is indicative of depolarization, whereas an increase is indicative of hyperpolarization [14]. As represented in Figure 3, compound was capable of significantly reducing mitochondrial membrane potential, promoting a depolarization of the mitochondrial membrane. Regarding *C. krusei*, compound reduced JC-1 ratio only when tested at the highest concentration (16 µg/mL, i.e., 2× MIC), significantly reducing JC-1 ratio to 84%, when compared with 100% of control. On the other hand, the compound promoted a mitochondrial membrane depolarization of *C. albicans* to all tested concentrations, an effect that occurred in a concentration-dependent manner, significantly decreasing JC-1 ratio values to 29.7%, 36.4% and 63.6% for 256 (2× MIC), 128 (MIC) and 64 (MIC/2) µg/mL, respectively, thus indicating the ability of compound **1** to significantly induce mitochondrial membrane depolarization in *C. albicans* (Figure 3). CCCP (100 μM) was used as a positive control for mitochondrial membrane depolarization, causing a significant reduction in JC-1 ratio to 39.9% and 26.1% in *Candida krusei* and *Candida albicans*, respectively (Supplementary Materials—Figure S1).

### 2.3. Effect on Intracellular ROS Levels Production

**Figure 4:**
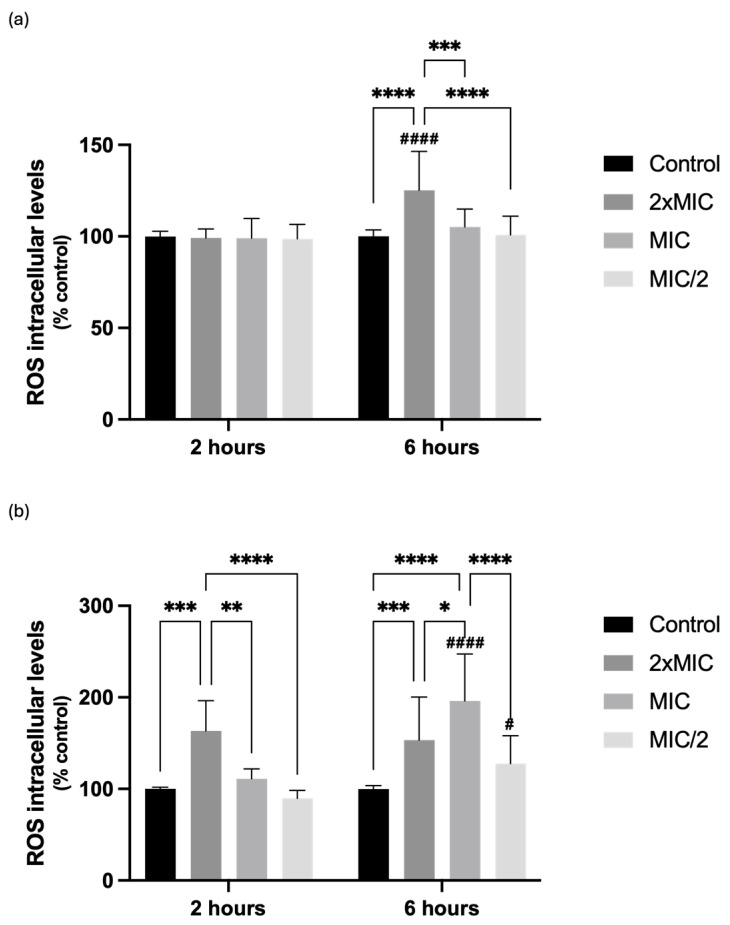
Effects of compound **1** on reactive oxygen species (ROS) production in (**a**) *Candida krusei* ATCC 6258 and (**b**) *Candida albicans* ATCC 10231 after 2 and 6 h of treatment. Results are presented as Mean + SD from 4 or 5 independent experiments, performed in duplicate [* *p* ˂ 0.05, ** *p* ˂ 0.01, *** *p* ˂ 0.001 and **** *p* ˂ 0.0001, for comparisons between concentrations at each timepoint; ^#^ *p* ˂ 0.05 and ^####^ *p* ˂ 0.0001, for comparisons between timepoints at each concentration]. MIC, minimal inhibitory concentration.

Regarding the effects of compound **1** on ROS production, the intracellular levels of ROS were evaluated after 2 and 6 h of exposure, in the two yeasts, by using the DCFH-DA probe. After 2 h of incubation, the compound did not promote any significant effects on *C. krusei* ROS intracellular levels, but significantly increased ROS intracellular levels on *C. albicans*, when tested at the highest concentration (ROS intracellular levels significantly increased to 163.5%, 2 h after exposure to 256 µg/mL, when compared with 100% of control yeasts) (Figure 4).

However, a significant increase on ROS intracellular levels was observed for *C. krusei* upon exposure for 6 h to the highest concentration of the compound (2× MIC, 16 μg/mL, 125.2%), while for *C. albicans*, significantly increased ROS levels were observed for 2× MIC and MIC (153.3% and 196.2%, respectively) (Figure 4). *t*-BHP (500 μM) was used as a positive control for ROS generation, causing a significant increase in ROS intracellular levels, both 2 h and 6 h after incubation, and for the two yeasts (Supplementary Materials—Figure S2 for results at 2 h and Figure S3 for results at 6 h).

### 2.4. Effect on Total ATP Intracellular Levels

**Figure 5:**
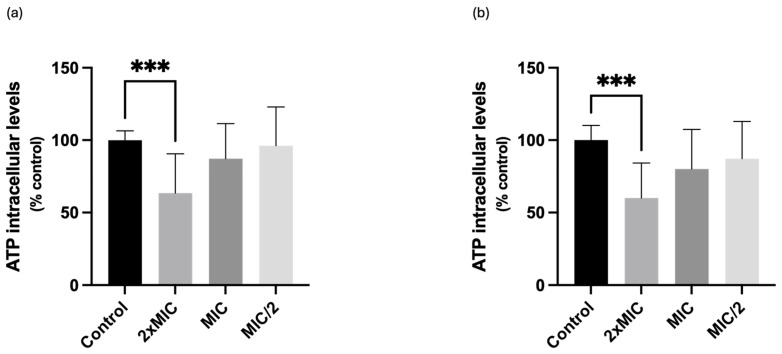
Effects of compound **1** on ATP intracellular levels in (**a**) *Candida krusei* ATCC 6258 and (**b**) *Candida albicans* ATCC 10231 cells. Results are presented as Mean + SD from 4 or 5 independent experiments, performed in duplicate. *** *p* < 0.001. MIC, minimal inhibitory concentration.

The effect of compound **1** on the intracellular ATP levels was also evaluated to more deeply understand if the depolarization of the mitochondrial membrane results in ATP depletion. For *C. krusei* and *C. albicans*, a significant decrease in ATP intracellular levels was observed for 2× MIC, which is in line with the results previously presented (depolarization of mitochondrial membrane and generation of ROS). In fact, in *C. krusei*, the compound significantly reduced the ATP intracellular levels to 63.4% (when compared with 100% of control yeasts), while in *C. albicans* it promoted a significant decrease of ATP intracellular levels to 60% (Figure 5).

### 2.5. Effect on the Viability of Human Cells

Compound **1** was evaluated at the screening program of CO-ADD for the elucidation of its possible cytotoxic effect against human cell lines. The CC_50_ value against the HEK-293 cell line and the HC_10_ value on human erythrocytes were of 1.1 and 0.8 μg/mL, respectively. Compound **1** was thus considered cytotoxic to human cells.

## 3. Discussion

The occurrence of fungal infections has seen a dramatically rise in recent years. Despite being often overlooked, serious mycoses afflict over 300 million individuals globally, resulting in an annual mortality rate of over 1.5 million [15]. The primary contributors to this mortality are pathogenic yeasts such as *Candida*, *Cryptococcus*, and *Pneumocystis*, as well as filamentous fungi like *Aspergillus*. Among these, *Candida* spp. are the most prevalent culprits for invasive mycoses [16]. *Candida albicans* stands out as the most common cause of candidiasis, though other *Candida* species also play significant roles in clinical scenarios, accounting for approximately 35–65% of candidemia cases. Notably, infections caused by *C. krusei* are recognized for their elevated mortality rates (40–58%) and limited response to standard antifungal treatments [17].

Cyclams are macrocyclic polyamines which have demonstrated medical interest, namely as antibacterial, antifungal and antiparasitic agents [18,19,20,21,22,23,24,25,26]. The cyclam salt H_4_[H_2_(^4-CF3^PhCH_2_)_2_Cyclam]Cl_4_ (compound **1**) was previously studied against *C. krusei* and *C. albicans*, and demonstrated a relevant antifungal activity, with minimal inhibitory concentration (MIC) values of 8 µg/mL for *C. krusei* and of 128 µg/mL for *C. albicans* [7]. More recently, this compound revealed to inhibit biofilm production and catalase activity, being able to interfere with *C. albicans* dimorphic transition [8]. Given those results, the potential mechanisms involved in the activity of this cyclam derivative were further studied.

The classic antifungal drugs used in therapy mainly target the cytoplasmic membrane by binding to ergosterol (polyenes) or by inhibiting ergosterol synthesis (azoles and allylamines). Later, a group of compounds with an action on the cell wall (echinocandins and a triterpenoid) has been introduced. The search for compounds with new targets of action or with multiple mechanisms of action is necessary in order to broaden activity, reduce toxicity and overcome the emergence of resistance. More recently, new compounds with different mechanisms have emerged, including fosmanogepix (an inhibitor of the Gwt1 enzyme) and olorophim (an inhibitor of the dihydroorotate dehydrogenase enzyme) [27]. Mitochondria is an essential target for the cell. Thus, compounds that act exclusively or partially on this target may show antifungal activity and be useful in the treatment of fungal infections [12].

For that purpose, and to understand if compound **1** had the ability to reduce the yeasts metabolic viability, REZ reduction assay was carried out. REZ solution is a blue-coloured solution, which shows little to no intrinsic fluorescence. When REZ diffuses through the cell membranes it is metabolically reduced by viable cells to the fluorescent to a pink-coloured product, resorufin. By measuring the fluorescence of this compound, it is possible to calculate the percentage of metabolic viable cells [28]. Accordingly, compound **1** had effect on both yeasts, significantly decreasing yeast metabolic activity, although this effect was more markedly observed on *C. albicans*. This reduction in metabolic activity suggests an impact on the organism’s ability to utilize alternative carbon sources and maintain normal cellular functions.

Mitochondria are critical for *Candida’s* metabolism, stress response, and virulence [10]. Therefore, three assays were performed to analyse the influence of compound **1** on the proper functioning of this organelle. In all assays, the compound demonstrated to interfere with *C. albicans* mitochondria. The compound significantly reduced mitochondrial membrane potential in both *C. albicans* and *C. krusei*, promoting its depolarization. Therefore, the observed effects point towards mitochondrial dysfunction, which can disrupt energy production (resulting in a reduction of ATP production), calcium balance, ROS production and cellular processes essential for the organism’s survival [14]. Accordingly, our results are in line with the literature, given that compound **1** promoted a significant increase in ROS production and a significant decrease in ATP intracellular levels in *C. albicans*, which agrees also with the observed decrease in mitochondrial membrane potential. The same happened for *C. krusei*, but in a lighter extent, which demonstrates a greater antifungal potential of this compound against *C. albicans*. The significant decrease in intracellular ATP levels caused by compound **1** thus clearly highlights its influence on energy metabolism in *C. albicans* and *C. krusei*. Furthermore, ATP is a critical energy source for cellular functions, and a decrease in ATP levels can impair various biological processes necessary for the organism’s viability [29].

Overall, compound **1’s** effects on *C. albicans* suggest a multi-faceted impact on the organism’s cellular functions. It significantly reduced metabolic activity (as seen through REZ reduction), disrupted mitochondrial function (evidenced by increased ROS production and mitochondrial membrane depolarization), and significantly decreased ATP levels, highlighting its influence on energy metabolism within the cells. The decreased sensitivity of *C. krusei* to compound **1** can be potentially justified by the distinct metabolic features of *C. krusei* and lower proteolytic potential when compared to *C. albicans*, which can impact its susceptibility to antifungal agents like compound **1** [30]. Furthermore, *C. krusei* exhibits differences in cell wall composition/structure compared to *C. albicans*, which can also influence its response to antifungal compounds like compound **1**. In addition, *C. krusei* is intrinsically resistant to fluconazole, both in vitro and in vivo [29,31,32]. This inherent resistance in *C. krusei* makes it more challenging to treat compared to other *Candida* species like *C. albicans*. Moreover, genetic determinants may also play a role in the resistance mechanisms of *C. krusei*, affecting its susceptibility to antifungal agents [33]. Variations in genetic factors between *Candida* species can thus potentially contribute to differences in resistance/sensitivity levels.

For an antimicrobial compound to be considered promising for therapeutic use, one of the parameters that is usually valued is the absence of toxicity to human cells [34,35]. Haemolytic activity in human erythrocytes is one of the most widely used tests for screening the cytotoxic activity of new bioactive compounds [34,35]. One of the problems with the haemolytic activity of these compounds is the limitation they impose on intravenous administration [36]. The erythrocyte membrane is a representative model of other cells, but there are studies which show that the sensitivity of erythrocytes to cytotoxicity may be greater than that of other human cells [34,35]. Moreover, the toxicity seen in in vitro tests is not always observed when in vivo tests are carried out [34]. In addition, one way of assessing the toxicity of compounds is to determine their effect on the metabolic viability of normal (i.e., non-tumour) cell lines [37,38]. One of the cell lines used for this purpose is the HEK-293 cell line (human kidney cells) [37,38]. Various strategies have been developed to avoid the haemolytic and cytotoxic activity of the compounds, such as their molecular modification [39] or the use of drug delivery systems, some already in clinical use and others being evaluated in clinical trials [40]. Amphotericin B is a good example of an antifungal drug widely used in the treatment of serious systemic infections, with a broad spectrum of action and a very low level of resistance, but which is very toxic to humans [41,42,43,44]. The toxicity associated with amphotericin B has implications for its clinical use, which is limited to inpatients due to the need, among other factors, for slow continuous intravenous infusion and the permanent need to monitor renal toxicity and body temperature [41,42,43,44]. For this reason, new therapeutic systems have been developed for administering this drug, and it continues to be used in therapy [41,42,43]. Also, to enable the use of nystatin for systemic use, different formulations are being tested [45]. As already stated, compound **1** has shown toxicity at the concentrations at which it is active as an antifungal. Therefore, molecular modifications or the development of drug delivery systems could be studied in order to increase its selectivity for the fungal cells and reduce its toxicity to humans. To reduce the concentrations to be used, synergism studies can be carried out using different compounds with antifungal activity. Associations of compounds with synergistic effects are a strategy to lower concentrations, toxicity and circumvent possible emergence of resistance, particularly if the compounds have different mechanisms of action. We foresee that incorporating the compound in drug nano-carriers, such as liposomes, polymer nanoparticles or inorganic nanoparticles that can be delivered specifically to the microbial cells may reduce cytotoxicity to the host. 

## 4. Materials and Methods

### 4.1. Standards and Reagents 

Dimethyl sulfoxide (DMSO), 3-(N-morpholino) propane-sulfonic acid (MOPS), resazurin (REZ), tert-butyl hydroperoxide (t-BHP), carbonyl cyanide m-chlorophenyl hydrazone (CCCP), luciferase from *Photinus pyralis* (firefly) and 2′,7′-dichlorofluorescin diacetate (DCFH-DA) were purchased from Sigma-Aldrich (St. Louis, MO, USA). RPMI-1640 broth medium (with L-glutamine, without bicarbonate and with the pH indicator phenol red) was purchased from Biochrom AG (Berlin, Germany). Phosphate buffered saline (PBS) was purchased from Fisher Reagent (Geel, Belgium). Potassium hydrogen carbonate (KHCO_3_), sodium dihydrogen phosphate dihydrate (Na_2_H_2_PO_4_.2H_2_O), adenosine 5′-triphosphate disodium salt hydrate and JC-1 were acquired from Thermo Fisher Scientific (Franklin, MA, USA). Perchloric acid 70% (HClO_4_) and Titriplex III (EDTA, disodium salt dihydrate) were obtained from Merck (Darmstadt, Germany). Sodium hydroxide (NaOH) was obtained from VWR (Fontenay-sous-Bois, France). D-Luciferin sodium salt was obtained from Abcam (Cambridge, United Kingdom).

Cyclam salt H_4_[H_2_(^4-CF3^PhCH_2_)_2_Cyclam]Cl_4_ (compound **1**) was synthesized according to a previously published procedure [22].

### 4.2. Effects on Yeasts’ Metabolic Activity—REZ Reduction Assay

The effect of the cyclam salt on yeasts metabolic viability was evaluated through the REZ reduction assay. Suspension of yeasts (*C. krusei* ATCC 6258 and *C. albicans* ATCC 10231) with a final cell density of 0.5–2.5 × 10^3^ CFU/mL were prepared in RPMI-1640 medium (RPMI-1640 with MOPS, pH 7.4), and incubated for 24 h at 37 °C. After this period of time, and using a 48-well plate, 990 µL of yeasts suspension were added in each well. Compound **1** was then prepared in RPMI-1640 medium to obtain the desired concentrations, and 10 µL was added to each well (final concentrations: 2× MIC, MIC and MIC/2), and incubated for 90 min at 37 °C. Yeasts were incubated with 10 µL of REZ prepared in RPMI-1640 medium (final concentration of 10 μg/mL) for 30 min at 37 °C. The fluorescence was measured using a multi-well plate reader (Synergy HT plate reader; BioTeck Instruments, Highland Park, IL, USA), using excitation and emission wavelengths of 560 nm and 590 nm, respectively. The effects of compound **1** on yeast’s metabolic activity was evaluated by the percentage of REZ reduction relatively to control yeasts. At least four independent experiments were performed in duplicate.

### 4.3. Effects on Yeasts’ Mitochondrial Membrane Potential—JC-1 Assay

The effect of compound **1** on yeast mitochondrial membrane potential was evaluated through the JC-1 assay [44]. Suspension of yeasts (*C. krusei* ATCC 6258 and *C. albicans* ATCC 10231) with a final cell density of 0.5–2.5 × 10^3^ CFU/mL were prepared in RPMI-1640 medium, and these working suspensions were placed into micro tubes (1 mL), and incubated for 24 h at 37 °C. Then, micro tubes were centrifuged at 15,493× *g* for 5 min. To the obtained cell pellet, compound **1** was added (at different concentrations and prepared in RPMI-1640 medium), and yeasts were further incubated for 2 h at 37 °C. Yeasts were then centrifuged (15,493× *g* for 5 min) and the obtained pellets were incubated for 30 min with 1 mL of JC-1 (10 µM) prepared in PBS. After this incubation period, cells were centrifuged at 15,493× *g*, for 5 min, and 500 µL of PBS were added to each pellet, and centrifuged again (in the same conditions). Finally, 500 µL of PBS were added to each pellet, and transferred to a 24-well plate. Fluorescence was measured in a multiwell plate reader (PowerWave-X, BioTek Instruments, Winooski, VT, USA) at 535 nm excitation and 595 nm emission wavelengths for JC-1 aggregates, and at 485 nm excitation and 535 nm emission wavelengths for JC-1 monomers. Results were analysed by the ratio between red and green fluorescence intensity, and expressed as percentage of control yeasts. At least four independent experiments were performed in duplicate. CCCP (100 µM, 2 h of incubation) was used as positive control for mitochondrial depolarization.

### 4.4. Effect on Intracellular ROS Level Production

To evaluate the effect of compound **1** on the production of ROS, suspension of yeasts (*C. krusei* ATCC 6258 and *C. albicans* ATCC 10231) with a final cell density of 0.5–2.5 × 10^3^ CFU/mL were prepared in RPMI-1640 medium, and these working suspensions were placed into micro tubes (1 mL), and incubated for 24 h at 37 °C. Then, micro tubes were centrifuged at 15,493× *g* for 5 min. To the obtained cell pellet, compound **1** was added (in different concentrations and prepared in RPMI-1640 medium) and incubated for 2 or 6 h at 37 °C. Yeasts were then centrifuged (15,493× *g* for 5 min) and the obtained pellets exposed to DCFH-DA (50 µM, prepared in PBS) transferred to a 48-well plate, and further incubated for 30 min. After this incubation period, the fluorescence was measured in a multiwell plate reader (PowerWave-X, BioTek Instruments, Winooski, VT, USA) using an excitation wavelength of 485 nm and an emission wavelength of 590 nm. The effects of the compound on intracellular ROS level production were expressed as percentage over control yeasts. At least four independent experiments were performed in duplicate. *t*-BHP was used as a positive control.

### 4.5. Effect on Total ATP Intracellular Levels

To evaluate the effect of compound **1** on intracellular ATP levels, suspension of yeasts (*C. krusei* ATCC 6258 and *C. albicans* ATCC 10231) with a final cell density of 0.5–2.5 × 10^3^ CFU/mL were prepared in RPMI-1640 medium. These working suspensions were placed into micro tubes (1 mL), and incubated for 24 h at 37 °C. Then, micro tubes were centrifuged at 15,493× *g* for 5 min. To the obtained yeasts pellets, compound **1** was added (in different concentrations and prepared in RPMI-1640 medium), and incubated for 2 h at 37 °C. After the incubation period, micro tubes were centrifuged for 5 min, at 250× *g*. Then, supernatant from each micro tube was discarded and ice-cold 5% HClO_4_ was added, followed by incubation of 30 min at 4 °C (for protein precipitation). After, the micro tubes were centrifuged at 15,493× *g* for 10 min. The acidic supernatants where then collected and stored at −80 °C until further determination of ATP content. The obtained cell pellets were resuspended in 1 M NaOH (overnight, at 4 °C), and then stored at −20 °C until being used for protein quantification.

For the determination of ATP intracellular levels, ATP standards solutions (0–16 μM) were prepared in 5% HClO_4_ for the creation of ATP calibration curves and stored at −80 °C until further use. The acidic supernatant samples and standards were neutralized with ice-cold 0.76 M KHCO_3_ (1:1) and mixed by vortex. The micro tubes were then centrifuged, for 10 min at 13,000 rpm (4 °C). All the procedures of samples and standards preparation were always performed on ice. Using a white 96-well plate, 75 μL of the neutralized supernatants of samples or standards were pipetted in duplicate. Already in the plate reader, 75 μL of the D-luciferin–luciferase solution [0.15 mM luciferin and 3,000,000 light unit’s luciferase/mL prepared in a 7.6 pH buffer solution (50 mM glycine; 10 mM MgSO_4_; 1 mM Trizma; 0.55 mM EDTA and 1% BSA)] were added, and bioluminescence immediately measured at 560 nm (28 °C), in a multiwell plate reader (PowerWave-X, BioTek Instruments, Winooski, VT, USA). *t*-BHP was used as a positive control. The ATP intracellular content in the samples was normalized to the protein content, which was quantified using the Bio-Rad DCTM protein assay kit. At least five independent experiments were performed in duplicate, and the results were expressed as percentage over control yeasts.

### 4.6. Cytotoxicity on Human Cells

Compound **1** was submitted to the screening program of the Community for Open Antimicrobial Drug Discovery CO-ADD [46] and its effect on the metabolic viability of the Human Embryonic Kidney 293 (HEK-293) immortalised cell line and its ability to cause the haemolysis of human red blood cells were evaluated. Briefly, and accordingly with CO-ADD information on their report, compound **1** was dissolved on DMSO (being 0.5% the maximum concentration used in the assays) and serial concentrations (1:2) were placed on the test plates (maximum concentration tested = 32 μg/mL and minimum concentration tested = 0.25 μg/mL). The interference with HEK-293 cell line metabolic viability was evaluated by the REZ reduction assay by adding this reagent at a concentration of 25 mg/mL, followed by a 3 h incubation on a CO_2_ (5%) incubator, at 37 °C. Fluorescence was read (excitation: 530/10 nm and emission: 590/10 nm; Tecan M1000 Pro monochromator plate reader) after the incubation period for compound **1** treated samples, negative control (free cell culture media) and positive control (cells without treatment). Results were expressed as concentration corresponding to 50% of toxicity (CC_50_) after designing a curve of inhibition versus log concentration values, and fitting those values using the function of Sigmoidal dose–response, with variable values (of top, bottom and slope), using the Pipeline-Pilot’s dose–response software (Dassault Systèmes, Version 18.1 (May 2018)). For the haemolytic assay the procedure was similar of that used for REZ and concentration at 50% haemolytic activity (HC_50_) was determined. The HC_10_ value was calculated using the following equation:

HC_10_ = HC_50_*(10/90)^(1/Slope). Compounds with CC_50_/HC_10_ ≤ maximum tested concentration were defined as having toxicity.

### 4.7. Statistical Analysis

GraphPad Prism 9 for MacOS (GraphPad Software, San Diego, CA, USA) was used for all statistical analysis. To perform the statistical comparisons between the tested conditions, one-way ANOVA was used, followed by the Dunnett’s multiple comparisons test. For experiments with two variables (ROS intracellular levels), two-way ANOVA followed by the Šídák’s multiple comparisons test was used. Details of the performed statistical analysis are described in the figure legend and, in all cases, *p* values smaller than 0.05 were considered significant.

## 5. Conclusions

Overall, the obtained data suggest that the exposure of both *C. albicans* and *C. krusei* to H_4_[H_2_(^4-CF3^PhCH_2_)_2_Cyclam]Cl_4_ resulted in a significant increase in ROS intracellular levels, accompanied by a significantly decreased metabolic activity and an impaired mitochondrial function. These effects collectively indicate a profound impact on the organism’s cellular functions, particularly on energy metabolism and overall cellular health.

## Data Availability

Not applicable.

## Supplementary Materials

The following supporting information can be downloaded at: https://www.mdpi.com/article/10.3390/ijms25105209/s1.
