# Supplementary material for: Disclosing the Antifungal Mechanisms of the Cyclam Salt H4[H2(4-CF3PhCH2)2Cyclam]Cl4 against Candida albicans and Candida krusei
Source: Int J Mol Sci. 2024 May 10;25(10):5209. doi: 10.3390/ijms25105209 (PMC11121207; doi:10.3390/ijms25105209)
Supplement: Supplementary file 1 [file ijms-25-05209-s001.zip › ijms-2961670-supplementary.pdf]

International Journal of
Molecular Sciences

Supplementary Material

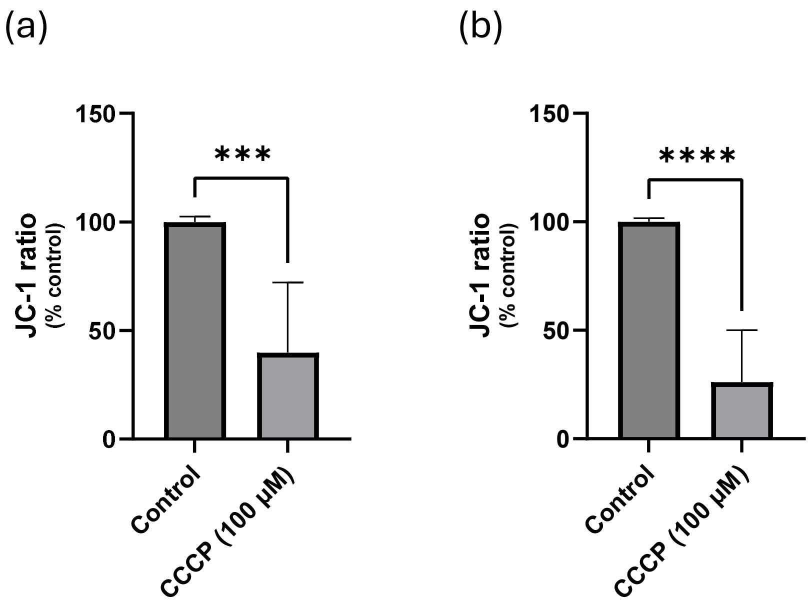

Figure S1. Effects of CCCP (100 µM, positive control) on the mitochondrial membrane potential (ΔΨm) of (a) *Candida krusei* ATCC 6258 and (b) *Candida albicans* ATCC 10231, evaluated using the JC-1 dye. Results are presented as Mean + SD from 4 independent experiments, performed in duplicate. *** $p < 0.001$; **** $p < 0.0001$.

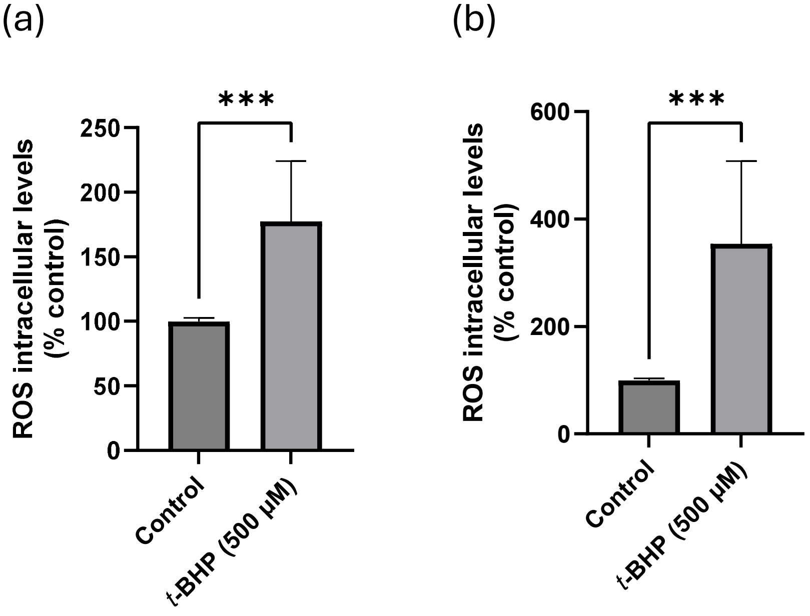

Figure S2. Effects of *t*-BHP (500 µM, positive control) on reactive oxygen species (ROS) production in *Candida krusei* ATCC 6258 after (a) 2 and (b) 6 hours of treatment. Results are presented as Mean + SD from 4 independent experiments, performed in duplicate. *** $p < 0.001$.

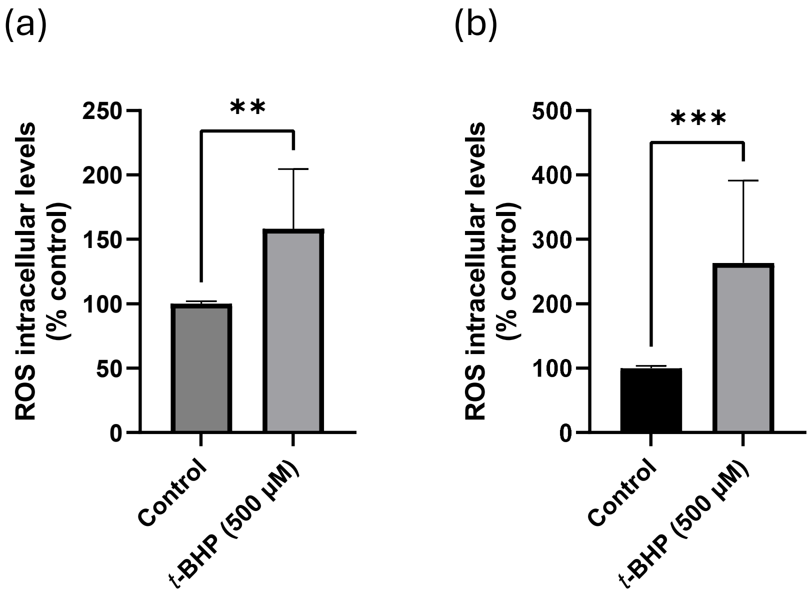

Figure S3. Effects of *t*-BHP (500 µM, positive control) on reactive oxygen species (ROS) production in *Candida albicans* ATCC 10231 after (a) 2 and (b) 6 hours of treatment. Results are presented as Mean + SD from 4 independent experiments, performed in duplicate. ** $p < 0.01$; *** $p < 0.001$.
